# A Bayesian latent class mixture model with censoring for correlation analysis in antimicrobial resistance across populations

**DOI:** 10.1186/s12874-021-01384-w

**Published:** 2021-09-20

**Authors:** Min Zhang, Chong Wang, Annette M. O’Connor

**Affiliations:** 1 Department of Statistics, Iowa State University, Ames, United States of America; 2 Department of Veterinary Diagnostic and Production Animal Medicine, Iowa State University, Ames, United States of America; 3 Department of Large Animal Clinical Sciences, Michigan State University, East Lansing, United States of America

**Keywords:** Correlation, Antimicrobial resistance, Minimum inhibitory concentration, Bayesian latent class model, NARMS

## Abstract

**Background:**

The emergence of antimicrobial resistance across populations is a global threat to public health. Surveillance programs often monitor human and animal populations to evaluate trends of emergence in these populations. Many national level antibiotic resistance surveillance programs quantify the proportion of resistant bacteria as a means of monitoring emergence and control measures. The reason for monitoring these different populations are many, including interest in similar changes in resistance which might provide insight into emergence and control options.

**Methods:**

In this research, we developed a method to quantify the correlation in antimicrobial resistance across populations, for the conventionally unnoticed mean shift of the susceptible bacteria. With the proposed Bayesian latent class mixture model with censoring and multivariate normal hierarchy, we address several challenges associated with analyzing the minimum inhibitory concentration data.

**Results:**

Application of this approach to the surveillance data from National Antimicrobial Resistance Monitoring System led to a detection of positive correlation in the central tendency of azithromycin resistance of the susceptible populations from *Salmonella* serotype Typhimurium across food animal and human populations.

**Conclusions:**

Our proposed approach has been shown to be accurate and superior to the commonly used naïve estimation by simulation studies. Further implementation of this Bayesian model could serve as a useful tool to indicate the co-existence of antimicrobial resistance, and potentially a need of clinical intervention.

## Background

### Introduction

Antimicrobial resistance (AMR) is a major threat to global public health for decades [1]. Surveillance programs form a critical part of the effort to identify and control the emergence of AMR. Knowledge of emerging resistance enables actions to mitigate the spread of emergence. For example, it was through surveillance systems that the emergence of ceftiofur resistance in poultry products, humans being associated with the introduction of the product into the poultry market, and the impact of antimicrobial elimination were detected [2]. Examples like these, which protect public health, illustrate the rationale for national AMR surveillance programs.

**Fig. 1:**
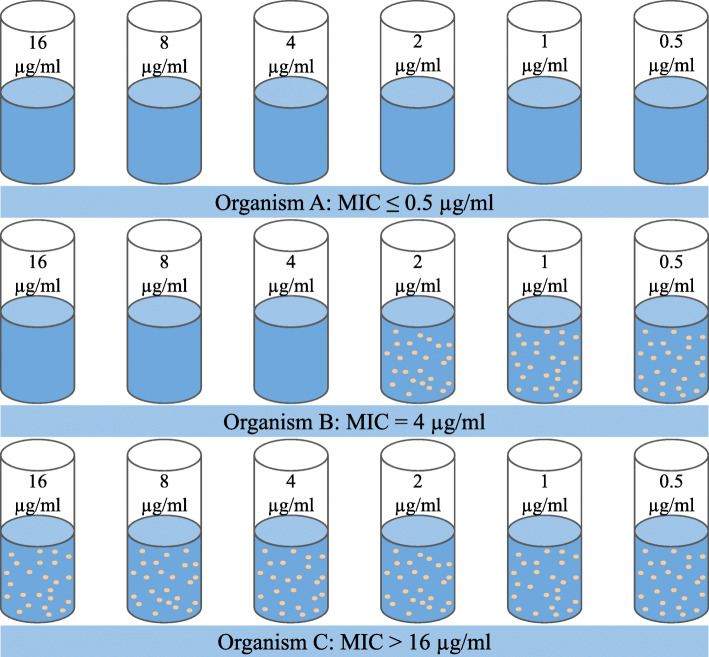
Determination of minimum inhibitory concentration (MIC) with the serial dilution method

In 1996, the National Antimicrobial Resistance Monitoring System (NARMS) was established to document the emergence of resistance from the use of antimicrobial drugs in the United States[3]. As a collaborative work between the Centers for Disease Control and Prevention (CDC), the U.S. Food and Drug Administration (FDA), and the United States Department of Agriculture (USDA), this national surveillance system tracks changes in the antimicrobial susceptibility of certain enteric bacteria found in ill people, retail meats, and food animals in the United States. This task is achieved by testing for the minimum inhibitory concentration (MIC), which is the lowest concentration of a particular antibiotic that will inhibit the bacteria growth (Fig. 1). MIC is currently measured from serial dilution experiments or obtained using the whole-genome sequencing based machine learning method [4].

Currently, data and analysis about AMR observed in NARMS are available in an integrated report. The predominant approach to quantifying antimicrobial resistance level is to use the proportion of isolates resistant to a particular antibiotic, which was defined as the number of isolates resistant to the antibiotic divided by the total number of isolates being tested [5]. However, comparative analyses of resistance across either serotypes, antibiotics, or populations are not included in the report, although such comparative analysis could provide additional insights into resistance emergence. For example, are patterns in resistance in one serotype of *Salmonella* correlated with those in other *Salmonella* serotypes? Or, are patterns in resistance observed in one *Salmonella* serotype correlated between human and food animal populations?

Existing research based on NARMS and other surveillance programs report that increased antimicrobial resistance in major food-borne and enteric bacteria (e.g. *Salmonella*, *Campylobacter*) has been found in human and food animal populations worldwide [6–8]. While many classes of antimicrobial agents used in food animals play important roles in growth enhancement and disease prevention, these data also raise the possibility of development of cross-resistance in human bacterial pathogens [9]. The ability to quantify the correlation in AMR level between human and food animal populations is an essential foundation for further studies on the transmission of antibiotic resistance across the populations. Positive correlations, which are of interest for antibiotic resistance surveillance data, refers to matched increases and/or decreases in a characteristic when compared across two or more populations. For MIC data, that characteristic could be either the resistance proportion or, a more interesting and comprehensive perspective, the mean MIC. The ability to evaluate multiple aspects of surveillance data certainly maximizes the value of society’s investment in such programs. In this paper, it is our focus to develop a methodology that gives accurate estimation of the correlation in AMR across populations using the mean MIC.

### Literature review

In NARMS surveillance data, isolates were classified into susceptible, intermediate, and resistant components based on their MIC relative to the breakpoints adopted from the Clinical and Laboratory Standards Institute (CLSI) [10]. With the understanding that an intermediate susceptibility to some drugs would remove this drug as a clinical option, isolates with intermediate susceptibilities could be considered as susceptible [11].

So far, the cross-population correlation in AMR has been analyzed with the popular characteristic of MIC data: the resistance proportion of an organism to an antibiotic. For example, Wegener [12] calculated the ceftiofur resistance proportion for retail chicken and human *Salmonella* Heidelberg isolates by using the moving average of quarterly proportion of resistance. A close AMR pattern between food supply and human was concluded in this work solely by visualization of their similar trends. A more recent example of correlation analysis in AMR level can be found in Iwamoto et al. [13], where Spearman rank correlation was used to examine the relationship between the annual proportion of ceftriaxone resistance among *Salmonella* isolates from human, retail meats, and food animals. However, percent resistant method relies on dichotomization of isolates (i.e. susceptible or resistant), hence losing information of the MIC distribution [14], and preventing monitoring of correlation in mean MIC. Additionally, breakpoints can vary across years, making direct comparison of proportions inappropriate. Finally, the use of the resistance proportions to correlation analysis is predicated on the presence of a resistant component, which is not always true.

Gradual movement or shift of mean MIC in susceptible population, a phenomenon referred to as MIC creep, has a different focus than changes in the resistance proportion or MIC geometric mean over a period of time [15]. In addition, correlation in mean MIC in susceptible population assists the identification of emerging joint resistance patterns. However, the challenge associated with the mean MIC estimation arises due to the censorship of the observations. For example, when observing an MIC of “=8” for an organism tested by some antibiotic, this MIC actually indicates that the true MIC is >4,≤8, and ultimately unknown. Estimation of the mean MIC based on unadjusted observations tends to be overestimated due to the upward rounding of data [16]. Another challenge related to the mean MIC estimation is the underlying population heterogeneity of the susceptible and resistant isolates. As indicated by the frequency plot of the observed MIC, it is natural to represent the true values with a bimodal distribution to reflect the two overlapping components (the susceptible bacteria being one component and resistant bacteria population being another component). Craig [17] resolved the censorship issue by integrating the uncertainty of the true lo*g*_2_MIC values in their underlying intervals and suggested modeling with a Gaussian mixture distribution. Under a different context, the isolates may also be classified into wild and non-wild components; and sometimes the non-wild component contains more than one component, which could be satisfied thanks to the flexibility of Gaussian mixture distribution. Subsequent research on estimating the full continuous scale MIC density with the semi-parametric [18] approach was conducted under Bayesian framework. Further analysis on the mean MIC creep was studied by Zhang et al. [19] with a linear model in the susceptible component by a fully parametric Bayesian method. Jaspers et al.[20] analyzed the joint distribution of MIC data on multiple antibiotics with Bayesian estimation of multivariate Gaussian mixtures, from which inference about the correlation between drug resistances within one year could be drawn. However, the multivariate means of MIC of each component were assumed as fixed from year to year, which ignores the potential for changes in the mean MIC for the components.

Evidence of cross-population correlation in the resistance proportion is available [13, 21]. Unlike the increase in the central tendency of the MIC for the susceptible population, an increase in the resistance proportion would most likely indicate the dissemination of less susceptible clone(s) [15]. Another important perspective is to look into the cross-population correlation in the movement of the MIC distribution, but to our knowledge it has not been studied yet. In this research, it is our interest to fill in the gap by estimating correlation in the mean MIC through a Bayesian framework with a multivariate normal hierarchy linking different populations.

### Contributions

In this paper, a Bayesian latent class mixture model with censoring and multivariate normal hierarchy was proposed to determine the correlation of the mean MIC of the susceptible isolates across two different populations. This mean MIC is unnoticed if only data about resistance proportions are used. The proposed model was applied to the datasets from National Antimicrobial Resistance Monitoring System (NARMS) obtained from CDC (human data) and USDA (food producing animal data). In order to obtain an estimation of the correlation in mean MIC across populations, we considered monthly means of lo*g*_2_MIC in the susceptible component and added a bivariate normal model in the hierarchical structure to evaluate the correlation in lo*g*_2_MIC between populations. In the example of *Salmonella enterica* Typhimurium tested by azithromycin in human and food animal datasets, a significantly positive correlation in the central shift of susceptible component was detected. Simulations on the proposed method were carried out for strong, moderate, and mild correlations, to show the precision of the new approach. Applications of our model to other organisms or antibiotics across populations could serve as evidences of emerging or declining joint resistance, adding value to already existing data collected by AMR surveillance programs.

## Methods

Our methodology estimates the correlation of the mean MIC for susceptible components across populations. In AMR surveillance programs, we observe MIC as the measurement of the antibiotic susceptibility of the isolates. Two-fold serial dilution data, like the MIC observations, are commonly analyzed with base 2 logarithm transformation. At the upper limit of the serial dilution where the growth of the bacteria could not be inhibited even with the highest drug concentration, the MIC is right censored. The resistant components are usually highly right censored. As a consequence, there is not enough information available to calculate the mean MIC for the resistant component of the data. Therefore, it is necessary to focus the correlation analysis on the susceptible components of bacteria.

In this section, a Bayesian latent class mixture model is introduced, and a straightforward analysis which we call the “naïve method” throughout this paper is used as a comparison.

### Model notations and assumptions

To fill the gap of estimating the cross-population correlation in the mean MIC of the susceptible component, we proposed a hierarchical Bayesian latent class mixture model and managed to address the challenges from the censored nature and the underlying distribution of the MIC data. To account for the censored nature, each observed MIC value was assumed to represent an interval where the true MIC value lies in.

The notations used are as follows:

- $y^{*}_{s,i,j}$: the observed value of lo*g*_2_MIC for isolate *j* in month *i* of population *s*.
- *y*_*s*,*i*,*j*_: the latent value of lo*g*_2_MIC for isolate *j* in month *i* of population *s*.
- *l*_*s*,*i*,*j*_,*u*_*s*,*i*,*j*_: the lower bound and upper bound of the latent true value *y*_*s*,*i*,*j*_, and *y*_*s*,*i*,*j*_∈(*l*_*s*,*i*,*j*_,*u*_*s*,*i*,*j*_]. Conversion between $y^{*}_{s,i,j}$ and the interval is defined in Table 1.

**Table 1:** Conversion table between the observed lo*g*_2_MIC ($y_{s,i,j}^{*}$) and the interval of its latent value (*y*_*s*,*i*,*j*_)

Observed $\mathrm {log_{2}MIC}: y_{s,i,j}^{*}$	Censor type	Interval of latent lo*g*_2_MIC
≤*a*_1_	Left censored	*y*_*s*,*i*,*j*_∈(−*∞*,*a*_1_]
=*a*_2_	Interval censored	*y*_*s*,*i*,*j*_∈(*a*_2_−1,*a*_2_]
>*a*_3_	Right censored	*y*_*s*,*i*,*j*_∈(*a*_3_,+*∞*)
- *c*_*s*,*i*,*j*_: the latent indicator of the bacterial component from which the isolate *j* in month *i* from population *s* was drawn. *c*=0,1 represents susceptible and resistant component, respectively.

The subscript *s*=1,2 represents the two populations whose correlation in the mean MIC are of interest to us; *i*=1,2,...,*I* is the time index, where *I* is the total number of months; *j*=1,2,...,*n*_*s*,*i*_ is the isolate index, where *n*_*s*,*i*_ is the number of observations for population *s* in month *i*. The definitions of the indices remain the same throughout the model description unless otherwise specified. In our example of the Real data analysis section, the populations across which the correlation was estimated were human and food animals.

### Model description

To estimate the correlation in the mean lo*g*_2_MIC in the susceptible component across populations, the Bayesian latent class mixture model with censoring and multivariate normal hierarchy is introduced in this section. In the data level, the latent true values of lo*g*_2_MIC from each population in each month are modeled by a Gaussian mixture distribution with Bernoulli distributed weights. This approach is motivated by the bimodal distribution of the frequency plots of the lo*g*_2_MIC observations. In the hierarchical structure of the model, a bivariate normal distribution (BVN) was imposed on the monthly means of lo*g*_2_MIC for the paired populations. The correlation parameter in the BVN distribution answers the question of whether the correlation in the mean MIC of the susceptible isolates exists across populations, hence is the most interesting parameter of the whole model.

The construction procedure of the proposed Bayesian hierarchical model that details the above structure is articulated below. For *s*=1,2; *i*=1,2,...,*I*; and *j*=1,2,...,*n*_*s*,*i*_:

1 $$ c_{s,i,j}|p_{s,i} \stackrel{ind}{\sim} Ber(p_{s,i}),  $$

2 $$  y_{s,i,j}|c_{s,i,j}, \beta_{0,s,i}, \beta_{1,s,i}, \sigma_{0,s}^{2}, \sigma_{1,s}^{2} \stackrel{ind}{\sim} \begin{cases} N(\beta_{0,s,i}, \sigma_{0,s}^{2}), c_{s,i,j}=0\\ N(\beta_{1,s,i}, \sigma_{1,s}^{2}), c_{s,i,j}=1\\ \end{cases}.  $$

Thev data model indicates that for population *s*, the true lo*g*_2_MIC of isolate *j* in month *i* is drawn from the resistant component with probability *p*_*s*,*i*_, and drawn from the susceptible component with probability 1−*p*_*s*,*i*_. Given that isolate *j* belongs to the susceptible component, the true value of its lo*g*_2_MIC follows a normal distribution with mean *β*_0,*s*,*i*_ and variance $\sigma _{0,s}^{2}$. Similarly, if that isolate *j* belongs to the resistant component, its lo*g*_2_MIC follows a normal distribution shifting to the right compared with the susceptible normal curve. The variance parameters of the Gaussian mixture model vary across component and population. But observations from the same population and component are expected to have the same spread. In the data model, the isolates are classified by a “soft” probabilistic threshold without reliance on the pre-determined breakpoints.

At this point, the data model depicts the distribution of lo*g*_2_MIC values separately for each month and each population. In fact, different sampling conditions (e.g. institution, technician, time, etc.) could cause heterogeneity in the measurement of the antibiotic susceptibility of the isolates. Therefore, it could be helpful to borrow information and integrate uncertainty in the mean lo*g*_2_MIC values across months via the hierarchical structure. In order to find evidence of association across populations, the model parameters from (1) and (2) are joined together through multivariate normal distributions:

3 $$  \text{logit}(p_{s,i}) =\text{log}\left(\frac{p_{s,i}}{1-p_{s,i}} \right) =\alpha_{s,i},  $$

4 $$  {}\boldsymbol{\alpha_{i}} \stackrel{iid}{\sim} MVN (\boldsymbol{\theta}, \boldsymbol{\Omega}), \: \text{where} \: \boldsymbol{\theta} = \left(\begin{array}{c} \theta_{1} \\ \theta_{2} \end{array}\right), \boldsymbol{\Omega} \,=\, \left(\begin{array}{cc} \tau_{1}^{2} & \eta\tau_{1}\tau_{2} \\ \eta\tau_{1}\tau_{2} & \tau_{2}^{2} \end{array}\right);  $$

5 $$  \begin{aligned} \boldsymbol{\beta_{0,i}} &\stackrel{iid}{\sim} MVN (\boldsymbol{\mu_{0}}, \boldsymbol{\Sigma_{0}}), \: \boldsymbol{\mu_{0}} = \left(\begin{array}{c} \mu_{0,1} \\ \mu_{0,2} \end{array}\right), \boldsymbol{\Sigma_{0}} \\&\quad= \left(\begin{array}{cc} \gamma_{0,1}^{2} & \rho_{0}\gamma_{0,1}\gamma_{0,2} \\ \rho_{0}\gamma_{0,1}\gamma_{0,2} & \gamma_{0,2}^{2} \end{array}\right); \end{aligned}  $$

6 $$ \begin{aligned} \boldsymbol{\beta_{1,i}} &\stackrel{iid}{\sim} MVN (\boldsymbol{\mu_{1}}, \boldsymbol{\Sigma_{1}}), \: \boldsymbol{\mu_{1}} = \left(\begin{array}{c} \mu_{1,1} \\ \mu_{1,2} \end{array}\right), \boldsymbol{\Sigma_{1}} \\&\quad= \left(\begin{array}{cc} \gamma_{1,1}^{2} & \rho_{1}\gamma_{1,1}\gamma_{1,2} \\ \rho_{1}\gamma_{1,1}\gamma_{1,2} & \gamma_{1,2}^{2} \end{array}\right). \end{aligned}  $$

Expressions (3) to (6) are the model’s hierarchical level, where the two populations are linked through a vector at a common time period and are modeled by bivariate normal distributions. In expression (4), ***α***_***i***_ refers to the vector of the resistance proportion of the two populations in month *i* after logit transformation: (*α*_*s*=1,*i*_,*α*_*s*=2,*i*_)^*T*^; boldface Greek letters are used to represent vectors. The vector of transformed proportions of resistance follows a multivariate normal distribution with mean vector ***θ*** and covariance matrix ***Ω***; capitalized boldface Greek letters are used to represent matrices. Since the amount of populations we study once at a time is two, the multivariate normal distribution is simply bivariate. In the case of *S* populations where *S*>2, the hierarchical structure could be extended to *S*-dimensional normal distributions.

It is important to remember that the objective of this paper is to find evidence of cross-population correlation in the monthly mean lo*g*_2_MIC, not considering the resistant isolates due to their heavy censorship. Expression (5) is about the susceptible component: ***β***_***0,i***_=(*β*_0,*s*=1,*i*_,*β*_0,*s*=2,*i*_)^*T*^, the vector of the mean lo*g*_2_MIC of two populations in month *i* is assumed to follow a BVN centered at (*μ*_0,1_,*μ*_0,2_)^*T*^; the parameters of the standard deviation *γ*_0,1_ and *γ*_0,2_ reflect the spread of the monthly mean lo*g*_2_MIC on the two dimensions of population. The correlation parameter *ρ*_0_ reflects the degree to which the lo*g*_2_MIC means of the susceptible isolates are linearly related across the populations, hence is the key to our research; *ρ*_0_∈[−1,1]. An estimation of *ρ*_0_ with small absolute value close to 0 indicates no or rather weak correlation in AMR across populations, while a large estimation close to 1 implies a strong positive correlation in AMR. When the latter scenario happens, our result could serve as an evidence of co-existence of emerging or declining AMR across populations. Similar with (5), expression (6) is an analog to the resistant component, but is of less interest to our study.

Consequently, the parameter space denoted as *Θ*, is (***σ***_***0***_,***σ***_***1***_,***θ***,***Ω***,***μ***_***0***_,***Σ***_***0***_,***μ***_***1***_,***Σ***_***1***_). To express the joint likelihood of the observations, we collapse all the observed and latent lo*g*_2_MIC in vectors ***y***^***∗***^ and ***y***, respectively. Let *f*(·|·) be generic expression of the conditional density. Then the joint likelihood of the observed lo*g*_2_MIC is written out as the joint likelihood of the latent lo*g*_2_MIC integrated over the intervals where the discrete observations lay in.

7 $$  {}f(\boldsymbol{y^{*}}|\Theta)= \int_{l_{2,I,n_{2,I} }}^{u_{2,I,n_{2,I} }} \cdots \int_{l_{1,1,1 }}^{u_{1,1,1 }} f(\boldsymbol{y}|\Theta) \, dy_{1,1,1} \cdots dy_{2,I,n_{2,I} }.  $$

As indicated by expressions (4) to (6), with the parameter space *Θ* given, the data parameters and *y*_*s*,*i*,*j*_ produced in month *i* are independent with those in month *i*^′^, where *i*≠*i*^′^. Hence, we have the joint likelihood of the latent lo*g*_2_MIC written as

8 $$  f(\boldsymbol{y}|\Theta) = \prod_{i=1}^{I} f(\boldsymbol{y_{i}}|\Theta),  $$

where ***y***=(***y***_***1***_,⋯,***y***_***I***_)^*T*^, and

9 $$ \begin{aligned} f(\boldsymbol{y_{i}}|\Theta)= \idotsint_{(\boldsymbol{\beta_{0,i}}, \boldsymbol{\beta_{1,i}}, \boldsymbol{\alpha_{i}})} & f(\boldsymbol{y_{i}}|\boldsymbol{\alpha_{i}},\boldsymbol{\beta_{0,i}},\boldsymbol{\beta_{1,i}},\boldsymbol{\sigma^{2}_{0}},\boldsymbol{\sigma^{2}_{1}}) f(\boldsymbol{\alpha_{i}}|\boldsymbol{\theta}, \boldsymbol{\Omega}) \\ & f(\boldsymbol{\beta_{0,i}}|\boldsymbol{\mu_{0}}, \boldsymbol{\Sigma_{0}}) f(\boldsymbol{\beta_{1,i}}|\boldsymbol{\mu_{1}}, \boldsymbol{\Sigma_{1}}) \,d\beta_{0,s_{1},i} \dots d\alpha_{s_{2},i} \end{aligned}  $$

The $f(\boldsymbol {y_{i}}|\boldsymbol {\alpha _{i}},\boldsymbol {\beta _{0,i}},\boldsymbol {\beta _{1,i}},\boldsymbol {\sigma ^{2}_{0}},\boldsymbol {\sigma ^{2}_{1}}) $ in equation (9) corresponds to data model level in expressions (1) - (2), and can be expressed as the product of the likelihoods of all *y*_*s*,*i*,*j*_ for *s*=1,2; *j*=1,...,*n*_*s*,*i*_. The *f*(***α***_***i***_|***θ***,***Ω***),*f*(***β***_***0,i***_|***μ***_***0***_,***Σ***_***0***_), and *f*(***β***_***1,i***_|***μ***_***1***_,***Σ***_***1***_) in equation (9) correspond to the hierarchical level of the model. According to the Bayes rule that *f*(*Θ*|***y***^***∗***^)∝*f*(***y***^***∗***^|*Θ*)×*f*(*Θ*), inference of the parameters needs to be drawn from the posterior distribution. The choice of prior distributions *f*(*Θ*) is explained in the following subsection.

### Prior distribution

Conjugate priors were assigned to the hyper mean parameters. We chose diffuse Gaussian priors for *θ*_1_,*θ*_2_∼*N*(0,10000) to reflect our lack of knowledge in the resistance proportion. But we chose moderately informative priors for *μ*_0,*s*_,*μ*_1,*s*_∼*N*(0,100) since we know that the magnitude of lo*g*_2_MIC could hardly be smaller than −10 or greater than 10. The restriction *μ*_0,*s*_<*μ*_1,*s*_ (*s*=1,2) was applied, because the susceptible mean should always be smaller than the resistant mean. A default uniform prior was assigned to the data standard deviation *f*(***σ***_***c***_)∝1 (*c*=0,1) in their positive spaces.

The selection of the prior for the covariance matrices is tricky. We adopted the separation strategy [22] over the popular inverse Wishart, since the latter distribution has a tendency to bias the posterior correlation downward [23]. Each of the covariance matrices was decomposed into a correlation matrix sandwiched by the scale matrices. The correlation matrix has 1’s on its diagonal and correlation parameters on its off-diagonal positions; the scale matrix has the standard deviation on the diagonal. For example, the covariance matrix of the monthly means of lo*g*_2_MIC in the susceptible component is written as:

10 $$  \begin{aligned} \boldsymbol{\Sigma_{0}} &= \left(\begin{array}{cc} \gamma_{0,1}^{2} & \rho_{0}\gamma_{0,1}\gamma_{0,2} \\ \rho_{0}\gamma_{0,1}\gamma_{0,2} & \gamma_{0,2}^{2} \end{array}\right)\\ & = \left(\begin{array}{cc} \gamma_{0,1} & 0 \\ 0 & \gamma_{0,2} \end{array}\right) \left(\begin{array}{cc} 1 & \rho_{0} \\ \rho_{0} & 1 \end{array}\right) \left(\begin{array}{cc} \gamma_{0,1} & 0 \\ 0 & \gamma_{0,2} \end{array}\right) \\ & =: \Gamma_{0} \mathrm{R}_{0} \Gamma_{0}. \end{aligned}  $$

Following the recommendation from the Stan development team [24], we used an LKJ prior for the correlation matrix [25], where *f*(R_0_)∝|R_0_|^*ν*−1^;*ν*>0. By choosing LKJ (*ν*=1) for the correlation matrices decomposed from *Ω*,*Σ*_0_, and *Σ*_1_, the densities of the priors are uniform over correlation matrices of their corresponding dimension *d* (in our case, *d*=2), reflecting our a priori lack of knowledge for the correlations. Weakly informative half-Cauchy priors were assigned to the scale parameters *τ*_*s*_,*γ*_0,*s*_,*γ*_1,*s*_∼Cauch*y*^+^(0,2);*s*=1,2. Here, all prior distributions were assumed independent.

### Naïve calculation of correlation

As a comparison with the proposed Bayesian method, we adopt the “naïve method”, a straightforward approach that can be found in the literature of correlation studies for MIC data of different drugs [26–29]. The naïve analysis for mean lo*g*_2_MIC ignores the nature of censoring of MIC data, and calculates the arithmetic average of lo*g*_2_MIC for the susceptible isolates within each month. For example, if an observed MIC value was =8 and was categorized as susceptible according to the CLSI standards, then we treated lo*g*_2_MIC=lo*g*_2_(8)=3 as the true lo*g*_2_MIC value and therefore used it for the naïve mean calculation for that month. For each population *s*, we could obtain a vector of monthly averages in the susceptible component over *I* months. The Spearman correlation coefficient between the vectors of means was used to describe the strength of correlation in the mean lo*g*_2_MIC of the susceptible isolates between the two populations. Hence, the name “naïve method” comes from the fact that this calculation does not take into account the censorship or the underlying distribution of the MIC data.

In the following Real data analysis and Simulation sections, we will implement both the Bayesian and the naïve methods to see how the data censorship and the mixture distribution affect their performances of estimating the cross-population correlation in the mean MIC of susceptible components.

## Real data analysis

### Data description and manipulation

#### Centers for disease control and prevention (CDC) human data

The human population of NARMS was launched in 1996 within the framework of CDC’s Emerging Infections Program and the Food-borne Diseases Active Surveillance Network (FoodNet)[5]. *Salmonella* isolates, as the largest genus type among the four bacteria in NARMS (others are *Campylobacter*, *Shigella*, and *Escherichia coli* O157), were reported with year of collection, serotype, MIC value tested against multiple antibiotics, the test conclusion (resistant or not), etc. We limited our analysis to an important serotype: *S.* Typhimurium. It has 5398 isolates, accounting for 14.1% of the 38311 *Salmonella* isolates, ranking second to Enteritidis (16.3%), and was tested for MIC for 28 antibiotics collected since 1996 till now.

#### U.S. department of agriculture (USDA) food producing animal data

The food animal component of NARMS includes data from 1997 to 2015 with monitoring of *Salmonella* and later expanded to *Campylobacter* (1998), *E. coli* (2000), and *Enterococcus* (2003)[30], in which *Salmonella* also forms the largest proportion of the data. Isolates are recovered from samples obtained at federally inspected slaughter and processing plants [5]. The information related to *Salmonella* Typhimurium included the MIC for 23 antibiotics, the month and year of collection, the host the isolate was obtained from, etc, but no test conclusion. There were 1161 *S.* Typhimurium isolates, accounting for 3.0% of the 38867 *Salmonella* isolates, ranking third after *Salmonella* Kentucky (9.3%) and *Salmonella* Enteritidis (4.1%).

#### Data manipulation

We selected *S.* Typhimurium treated by azithromycin (AZI) as an example for illustration. AZI, a clinically important macrolide antibiotic, is used to treat a wide variety of bacterial infections, and is often for the treatment of nontyphoidal *Salmonella* (NTS) when treatment is indicated [31]. According to the NARMS integrated report of 2015 [32], AZI use for NTS is increasing, likely caused by the concerns about resistance to fluoroquinolones (e.g., ciprofloxacin) [33]. In livestock, macrolides are a commonly used antibiotic for treatment and control of disease, especially in cattle and swine, where they are highly effective for common diseases such as respiratory disease.

The pairs of the mean lo*g*_2_MIC during common time periods between human’s and food animals’ susceptible components allow us to calculate their cross-population correlation. Using monthly means of lo*g*_2_MIC, instead of yearly means, provides us with more pairs of data, hence it is beneficial for correlation calculation. For this reason, we acquired, with permission, from CDC the month of isolate collection which is not publicly available.

**Fig. 2:**
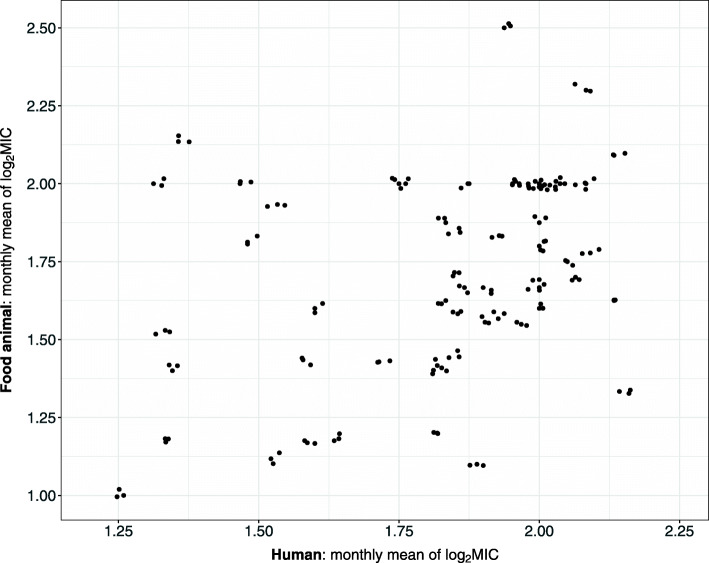
Jittering scatter plot of the monthly arithmetic mean lo*g*_2_MIC of susceptible *Salmonella* Typhimurium isolates tested by azithromycin in human and food animal populations from January 2011 to December 2015

At the time of this work, NARMS data after year 2017 are preliminary and the isolate collection and/or testing are still in progress, thus not included in our analysis. Since the AZI test for *Salmonella* started officially in 2011 [32], the trial samples occurring before 2011 were removed; this represented removal of 25 human isolates and 20 food animal isolates collected in 2008. For the cases where the month information or the MIC results of AZI testing are missing, the isolates were excluded from our analysis. After such elimination, 1333 human isolates from 2011 to 2017 and 572 food animal isolates from 2011 to 2015 remained, leading to 60 pairs of monthly mean of lo*g*_2_MIC for the susceptible component spanning from January 2011 to December 2015. On average, there were 16 human observations and 10 food animal observations per month during this five-year period. The scatter plot of the monthly arithmetic mean lo*g*_2_MIC of susceptible isolates is displayed in Fig. 2 for human and food animal populations from January 2011 to December 2015, where a moderate cross-population correlation in mean lo*g*_2_MIC could be seen.

In the CDC human data, the test conclusion for *Salmonella* AZI used the cutoff of >16 *μ*g/ml between 2011 and 2015, and ≥ 32 *μ*g/ml for the other years. In the USDA food animal data, *Salmonella* isolates tested by AZI were right censored at >16 *μ*g/ml consistently through all years, but the test conclusions are not included in the dataset. We adopted >16 *μ*g/ml as the cutoff rule for our example data during 2011 to 2015, even though it is stated in the NARMS integrated report [32] that *Salmonella* isolates from humans, retail meats, and food animals used the CLSI investigational breakpoint of ≥ 32 *μ*g/ml in order to determine susceptibilities to AZI. This is an example of inconsistent breakpoint and dilution spectrum. Based on the data manipulation described above and the breakpoint of >16 *μ*g/ml, there was only one azithromycin-resistant isolate in each of the two populations during this period. This is a very low level of resistance, leaving the analysis based on resistance proportion impossible. For situations like this, assessing the correlations in the mean MIC of the susceptible isolates (major component) could add useful information for the decisions makers.

### Implementation

To draw inference from the proposed model, a Bayesian analysis of Markov Chain Monte Carlo (MCMC) was conducted in R environment (version 4.0.0) with package *rstan* [34]. To sample effectively from the posterior *f*(*Θ*|***y***^***∗***^) that has no closed form, No-U-Turn Sampler (NUTS) [35], an extension of the powerful Hamiltonian Monte Carlo (HMC), was implemented; it is also the default and preferred sampling algorithm in Stan. All relevant R scripts, including cleaning of the NARMS data, model construction, and model implementation can be found on a public GitHub repository [36].

The choice of the initial values of the MCMC was based upon the selected example dataset of *S.* Typhimurium tested by AZI from NARMS. For population *s*, the initial values of the variances $\sigma ^{2}_{c,s}$ in equation (2) were calculated by the variance of all observed lo*g*_2_MIC values in component *c*, regardless of the month the isolate was obtained. The initial value of the monthly resistance proportion after logit transformation for population *s* (*α*_*s*,*i*_) was calculated by dividing the number of resistant isolates with the total number of isolates in that month followed by logit transformation. When all isolates appeared to be under or above the breakpoint in some months, a very small proportion was added to 0 or deducted from 1, to make sure $\alpha _{s,i}=\text {log}(\frac {p_{s,i}}{1-p_{s,i}})$ are finite values. The starting point of the monthly mean of lo*g*_2_MIC for population *s* in component *c* (*β*_*c*,*s*,*i*_) was calculated as the arithmetic average of the observed lo*g*_2_MIC in the corresponding subset of data. If in some months, one component has no observation, then these were imputed by the average of the other monthly means in the same component of that population.

The initial values of the above *α*_*s*,*i*_,*β*_*c*=0,*s*,*i*_, and *β*_*c*=1,*s*,*i*_ can be arranged as *I*×2 data frames with the row representing month and the column representing population. The means over the time index (i.e. column means of the data frames) were used as the initial values of the bivariate mean vectors ***θ***,***μ***_***0***_ and ***μ***_***1***_ in equations (4), (5) and (6). Similarly, the standard deviations over month (i.e. column standard deviations of the data frames) were assigned as the initial values of the scale parts of matrices ***Ω***,***Σ***_***0***_ and ***Σ***_***1***_. The Spearman correlations across populations were entered into the correlation matrices as starting values.

For the MCMC, we conducted three chains simultaneously with ten thousand iterations and 50% of the length was burn-in. We used the potential scale reduction factor of Gelman and Rubin [37] to assess convergence of the three chains. Additionally, we calculated the number of effective samples for each sampled parameter to ensure reasonable accuracy in the tails of the posterior distribution. The point estimates of the model parameters were determined by the means of the posterior draws after the burn-in sessions. The ends of the 95% credible intervals (CI) were obtained from the 2.5th and 97.5th percentiles of the posterior distributions. The results were summarized in the following Results Section.

## Results

The correlation in the mean lo*g*_2_MIC of the susceptible *S.* Typhimurium isolates tested by azithromycin across the human and food animal populations was estimated through the application of the proposed Bayesian latent class mixture model with censoring and multivariate normal hierarchy on the NARMS datasets from January 2011 to December 2015. The Bayesian estimation of the correlation parameter, its 95% credible interval, and the estimation through the naïve method are listed in Table 2.

**Table 2:** Correlations in susceptible lo*g*_2_MIC across populations for *Salmonella enterica* Typhimurium isolates estimated by Bayesian and naïve methods

Population 1	Population 2	Bayesian estimation	CI of Bayesian method	Naïve estimation
human	animal	0.4600	(0.1017, 0.7583)	0.3688

The point estimation of 0.46 together with its 95% credible interval (0.1017,0.7583) from the Bayesian approach indicate that there exists a significant positive correlation in the mean lo*g*_2_MIC between the susceptible isolates in the human population and those in the food animal population. It implies that the conventionally unnoticed MIC creep occurred in these two populations moved in similar directions. Interestingly, we found that the Bayesian point estimation is greater than the naïve estimate by 0.0912. It is conjectured that the estimation from the proposed Bayesian approach is more accurate compared with the naïve method, because the former one takes into account the censorship issue and makes full use of the data information. To validate this conjecture, a simulation study was conducted and the performance of the two methods were compared.

## Simulation

In this section, a simulation study was conducted to assess the performance of the proposed hierarchical Bayesian latent class mixture model and the naïve method, by comparing their estimation results with the underlying data generators.

In the following description of data simulation, we denote the known model parameters (***σ***_***c***_,***θ***,***μ***_***c***_,***Ω***,***Σ***_***c***_; *c*=0,1) with a “hat” on top of the Greek letters. These parameters were given the pre-determined values that were estimated from the application of the Bayesian approach on the human-food animal example in the Real data analysis section, so that the simulated datasets are close to what we might observe in the real world. In particular, we are most interested in the correlation parameter $\hat {\rho }_{0}$, which is the correlation part of ***Σ***_***0***_, was given the true value of 0.46. In each simulated dataset, there are 60 months, 16 human isolates and 10 animal isolates per month; this is to ensure a similar size of observations with the real dataset.

For *s*=1,2; *i*=1,2,...,*I*; *j*=1,2,...,*n*_*s*,*i*_; where *I*=60,*n*_*s*=1,*i*_=16 and *n*_*s*=2,*i*_=10:

(i) Generate $\boldsymbol {\alpha _{i}} = \left (\alpha _{s=1,i}, \alpha _{s=2,i} \right)^{T} \stackrel {iid}{\sim } MVN_{2} (\boldsymbol {\hat {\theta }}, \boldsymbol {\hat {\Omega }})$.
(ii) Convert ***α***_***i***_ to ***p***_***i***_, the vector of monthly proportion of resistant isolates, through expit transformation (inverse of logit): $\boldsymbol {p_{i}} = \left (p_{s=1,i}, p_{s=2,i} \right)^{T} = \frac {1}{1+exp^{-\boldsymbol {\alpha _{i}} }}$.
(iii) Generate the vector of monthly mean lo*g*_2_MIC in the susceptible and resistant population, respectively:
  $\boldsymbol {\beta _{0,i}} = \left (\beta _{c=0,s=1,i}, \beta _{c=0,s=2,i} \right)^{T} \stackrel {iid}{\sim } MVN_{2} (\boldsymbol {\hat {\mu }_{0}}, \boldsymbol {\hat {\Sigma }_{0}})$;
  $\boldsymbol {\beta _{1,i}} = \left (\beta _{c=1,s=1,i}, \beta _{c=1,s=2,i} \right)^{T} \stackrel {iid}{\sim } MVN_{2} (\boldsymbol {\hat {\mu }_{1}}, \boldsymbol {\hat {\Sigma }_{1}})$.
(iv) Generate the latent variable of class indicator for each population *s* and month *i*: *c*_*s*,*i*,*j*_∼*indBer*(*p*_*s*,*i*_).
(v) Generate the latent value of lo*g*_2_MIC for each population *s* and month *i*:
  $$ \begin{aligned} y_{s,i,j} \stackrel{ind}{\sim} \left\{ \begin{array}{ll} N(\beta_{c=0,s,i}, \sigma_{0,s}^{2}), c_{s,i,j}=0\\ N(\beta_{c=1,s,i}, \sigma_{1,s}^{2}), c_{s,i,j}=1\\ \end{array} \quad. \right. \end{aligned} $$
  The lo*g*_2_MIC obtained from step (v) are continuous quantities drawn from a Gaussian mixture model and need to be censored by following the conversion rule described in Table 1. Step (vi) describes the censoring operation for the example of *S.* Typhimurium tested by azithromycin.
(vi) Convert the latent value of lo*g*_2_MIC*y*_*s*,*i*,*j*_ to the censored value $y^{*}_{s,i,j}$:
  $$\begin{aligned} y^{*}_{s,i,j} = \left\{ \begin{array}{lll} -2, y_{s,i,j}\le -2 \\ {\lceil}{y_{s,i,j}}{\rceil}, -2 < y_{s,i,j} \le 4\\ 4, y_{s,i,j} > 4\\ \end{array} ; {\lceil}{\cdot}{\rceil} \text{represents the ceiling of a number.} \right. \end{aligned} $$
  According to the dilution spectrum we observed from the application data, the most susceptible isolates were from human samples with MIC = 0.5 *μ*g/ml. It indicates that if *y*_*s*,*i*,*j*_≤log_2_(0.5)−1=−2, it will be left censored as $y^{*}_{s,i,j}\le -2$; that is *l*_*s*,*i*,*j*_=−*∞* and *u*_*s*,*i*,*j*_=−2. The most resistant isolates came from human and food animal samples with MIC greater than 16 *μ*g/ml. Similarly, it means that if *y*_*s*,*i*,*j*_>log_2_(16)=4, it will be right censored as $y^{*}_{s,i,j}>4$; that is *l*_*s*,*i*,*j*_=4 and *u*_*s*,*i*,*j*_=+*∞*. A latent value of lo*g*_2_MIC in between -2 and 4 will be interval censored with its upper bound being the nearest integer to the right and lower bound being the nearest integer to the left.

End of simulation.

By repeating the above procedure 100 times, we obtained 100 simulated datasets, each of which was used to estimate correlation with the proposed Bayesian model and the naïve method. The two approaches were assessed and compared by the mean bias of *ρ*_0_, and its root of mean squared error (RMSE). These two metrics are the indicators of a model’s precision by measuring the average bias to the truth and the average deviation around the truth. Since it is important to assess the model performance under different strengths of correlation, we also conducted simulations for $\hat {\rho }_{0}=0.8$ (strong correlation) and $\hat {\rho }_{0}=0.3$ (weak correlation) with the other settings unchanged. The mean estimation bias and RMSE from the simulations can be found in Table 3.

**Table 3:** Simulation results based on different true values of correlations, estimated by Bayesian and naïve methods

True correlation	Method	Mean estimation bias	Root of mean squared error
0.80	Bayes	−0.0219	0.0927
	Naïve	−0.1972	0.2134
0.46	Bayes	−0.0025	0.1461
	Naïve	−0.1002	0.1510
0.30	Bayes	+0.0115	0.1621
	Naïve	−0.0561	0.1360

## Discussion

When estimating the cross-population correlation in the mean lo*g*_2_MIC of the susceptible isolates, the proposed Bayesian latent class mixture model shows advantages compared with the naïve method. In the simulation results (Table 3), the absolute values of the mean estimation bias of the Bayesian method are small compared with the scale of lo*g*_2_MIC data, and are much smaller than those of the naïve method. By comparing the RMSE, we can find that the Bayesian method has much smaller deviation from the true value for the strong correlation case. For moderate and weak correlations, the variation in error of the two methods are comparable since their RMSE are of similar scale. Overall, the proposed method is reasonably accurate and superior to the naïve method for estimating correlation.

According to Annis and Craig [16], neglecting the censored nature of the MIC data in the naïve method leads to overestimation in the mean of lo*g*_2_MIC, while they did not investigate its impact in correlation. Intuitively, when the two populations are positively correlated in the monthly mean of the latent lo*g*_2_MIC, the inherent correlation could be dwarfed by the upward rounding procedure, as shown by the underestimation of the naïve method in the simulation study. This points to the importance of the censorship adjustment included in the Bayesian model.

Another superiority of the Bayesian latent class mixture model lies in the independence of the methods from the breakpoint value. The naïve method subsets the dataset by relying on the NARMS-established breakpoints and considering only the susceptible isolates under the cutoffs. It is not a rare case where the CLSI breakpoints were updated for some antimicrobial agents in the NARMS history. For example, CDC Human’s *Salmonella* resistance to streptomycin was adjusted from ≥ 64 *μ*g/ml to ≥ 32 *μ*g/ml in 2014, hence the current breakpoint could not be applied to the previous years due to limited concentrations tested [10]. Apart from this, Mouton [38] argued that there is a major difference between clinical and microbiological breakpoints, where the former is an indicator for clinical success while the latter is for detecting resistant populations. Therefore, despite the simplicity of the naïve method, it has obvious defects compared with the proposed hierarchical Bayesian model.

Several assumptions were made in our model. We assumed normal distributions for susceptible and resistant components, as confirmed by the distributions of the observed lo*g*_2_MIC in the application and other examples. We found that many of the cases in the NARMS datasets present two components. However, when this is violated by single or more than two components, the latent variable mixture modeling approach becomes problematic in deciding the number of components and is considered as a limitation of this method [39]. In this situation, an alternative model with appropriate number of clusters can be developed instead.

In summary, the proposed Bayesian latent class mixture model addresses the challenges associated with the MIC data analysis, and provides accurate estimation of correlation in mean lo*g*_2_MIC of susceptible isolates across populations. It performs especially well when the true correlation is strong, which is crucial to public health as co-resistance of antibiotics across populations could signal needs of remedial actions.

## Conclusions

In this work, we proposed a Bayesian latent class mixture model with censoring and multivariate normal hierarchy for inference of the correlation in antimicrobial resistance across populations. Besides the cross-population correlation on the resistance proportion which has been the focus in the existing literature, we also targeted the correlation in the mean MIC of the susceptible populations. By applying the model to the NARMS data, we detected positively correlated mean MIC shift in the susceptible component between human and food animal populations. This means that for the susceptible isolates, the monthly MIC means in human and in food animal are changing more in the same direction than in irrelevant directions. This result indicates a possibility for the introduction of azithromycin resistance of *S.* Typhimurium in human through food animals, which warrants further investigation.

In future work, we are going to study several extended questions about the correlated antimicrobial resistance. One aspect is to evaluate the correlation in the mean MIC across antibiotics, which could help provide evidence of co-resistance among drugs. Another aspect is to see whether the mean MIC for an antibiotic in two serotypes are positively correlated. If the answer is yes and the serotypes possess different resistance gene, this suggests the genes share a similar mechanism of inducing resistance. The ability to assess correlations from more perspectives would enable increased information to be extracted from the AMR surveillance programs, and create further value to the public health.

## Data Availability

All relevant R scripts are available in a public GitHub repository [36]. The CDC human data that support the findings of this study (with month of isolation) are not publicly available due to privacy restrictions, but are available on request from CDC. The USDA food producing animal data were derived from NARMSIntegratedReports/Summaries [40].

## Abbreviations

AMR: Antimicrobial resistance
NARMS: National Antimicrobial Resistance Monitoring System
CDC: Centers for Disease Control and Prevention
FDA: U.S. Food and Drug Administration
USDA: United States Department of Agriculture
MIC: Minimum inhibitory concentration
CLSI: Clinical and Laboratory Standards Institute
BVN: Bivariate normal distribution
FoodNet: Foodborne Diseases Active Surveillance Network
AZI: Azithromycin
NTS: Nontyphoidal *Salmonella*
MCMC: Markov Chain Monte Carlo
NUTS: No-U-Turn Sampler
HMC: Hamiltonian Monte Carlo
CI: Credible interval
RMSE: Root of mean squared error
